# Cysteinyl Leukotriene Signaling Aggravates Myocardial Hypoxia in Experimental Atherosclerotic Heart Disease

**DOI:** 10.1371/journal.pone.0041786

**Published:** 2012-07-25

**Authors:** Elena Nobili, M. Dolores Salvado, Lasse Folkersen, Laura Castiglioni, Jens Kastrup, Anders Wetterholm, Elena Tremoli, Göran K. Hansson, Luigi Sironi, Jesper Z. Haeggström, Anders Gabrielsen

**Affiliations:** 1 Monzino Cardiologic Center, IRCCS, Milan, Italy; 2 Department of Pharmacology, University of Milan, Milan, Italy; 3 Department of Medical Biochemistry (MBB) II, Karolinska Institutet, Stockholm, Sweden; 4 Department of Cardiology B, Cardiac Catheterization Laboratory, Rigshospitalet, Copenhagen, Denmark; 5 Department of Medicine, Exp. Cardiovascular Research, Center for Molecular Medicine, Karolinska Institutet, Stockholm, Sweden; Instituto de Biofisica Carlos Chagas Filho, Universidade Federal do Rio de Janeiro, Brazil

## Abstract

**Background:**

Cysteinyl-leukotrienes (cys-LT) are powerful spasmogenic and immune modulating lipid mediators involved in inflammatory diseases, in particular asthma. Here, we investigated whether cys-LT signaling, in the context of atherosclerotic heart disease, compromises the myocardial microcirculation and its response to hypoxic stress. To this end, we examined Apoe^−/−^ mice fed a hypercholesterolemic diet and analysed the expression of key enzymes of the cys-LT pathway and their receptors (CysLT1/CysLT2) in normal and hypoxic myocardium as well as the potential contribution of cys-LT signaling to the acute myocardial response to hypoxia.

**Methods and principal findings:**

Myocardial biopsies from Apoe^−/−^ mice demonstrated signs of chronic inflammation with fibrosis, increased apoptosis and expression of IL-6, as compared to biopsies from C57BL/6J control mice. In addition, we found increased leukotriene C_4_ synthase (LTC_4_S) and CysLT1 expression in the myocardium of Apoe^−/−^ mice. Acute bouts of hypoxia further induced LTC_4_S expression, increased LTC_4_S enzyme activity and CysLT1 expression, and were associated with increased extension of hypoxic areas within the myocardium. Inhibition of cys-LT signaling by treatment with montelukast, a selective CysLT1 receptor antagonist, during acute bouts of hypoxic stress reduced myocardial hypoxic areas in Apoe^−/−^ mice to levels equal to those observed under normoxic conditions. In human heart biopsies from 14 patients with chronic coronary artery disease mRNA expression levels of LTC_4_S and CysLT1 were increased in chronic ischemic compared to non-ischemic myocardium, constituting a molecular basis for increased cys-LT signaling.

**Conclusion:**

Our results suggest that CysLT1 antagonists may have protective effects on the hypoxic heart, and improve the oxygen supply to areas of myocardial ischemia, for instance during episodes of sleep apnea.

## Introduction

Chronic ischemic heart disease is characterized by inadequate oxygen supply to the myocardium at rest or in response to increased demand and is usually caused by flow restriction with normal blood oxygen content. However, under certain circumstances reduction in oxygen supply due to decreased blood oxygen content may be the main eliciting cause of inadequate oxygen supply to the myocardium, as for example observed during sleep apnea.

Patients with sleep apnea exhibit a variant number of reductions and severity of decreased blood oxygen content during sleep. Notably, systemic leukotrienes are increased in patients with obstructive sleep apnea and increased LTB_4_ signaling has been suggested to contribute to development of atherosclerotic lesions [1], [2], [3], [4], [5]. Furthermore, increased cysteinyl leukotriene (cys-LT) signaling has been shown to cause vasoconstriction in atherosclerotic coronary vessels [6], suggesting an important role for these mediators in the cardiovascular response to sleep apnea.

It is therefore possible that during bouts of hypoxia, such as those seen with sleep apnea disorders, the heart begins to produce leukotrienes [5], [7], pro-inflammatory molecules derived from the 5-lipoxygenase (5-LO) pathway of arachidonic acid metabolism [8]. Exogenous cys-LTs elicit severe vascular constriction of the coronaries and cardiac arrhythmias during intracoronary administration [9] and cys-LT signaling can aggravate myocardial ischemia-reperfusion injury and infarction, as well as impede remodeling after myocardial injury in certain experimental models [10], [11]. It is possible that vasoconstrictor and/or pro-inflammatory effects of chronic and acute-in-chronic cys-LT signaling “per se” aggravate myocardial hypoxia and inflammation during bouts of hypoxia and would be particularly detrimental in the setting of chronic ischemic heart disease.

Such a notion would require that 1) the constitutional components of the cys-LT pathway are expressed in the myocardium and 2) that interruption of cys-LT signaling during bouts of hypoxia is beneficial. Accordingly, we investigated the gene expression and role of the cys-LT signaling cascade in a mouse model of atherosclerotic heart disease and compared the results with gene expression data from specimens of human myocardium. Specifically, we tested the hypothesis that cys-LT signaling plays a role in sustaining myocardial ischemia during bouts of hypoxia in chronic ischemic heart disease.

## Results

### The Apoe^−/−^ Mouse as a Model of Chronic Ischemic Heart Disease

**Figure 1:**
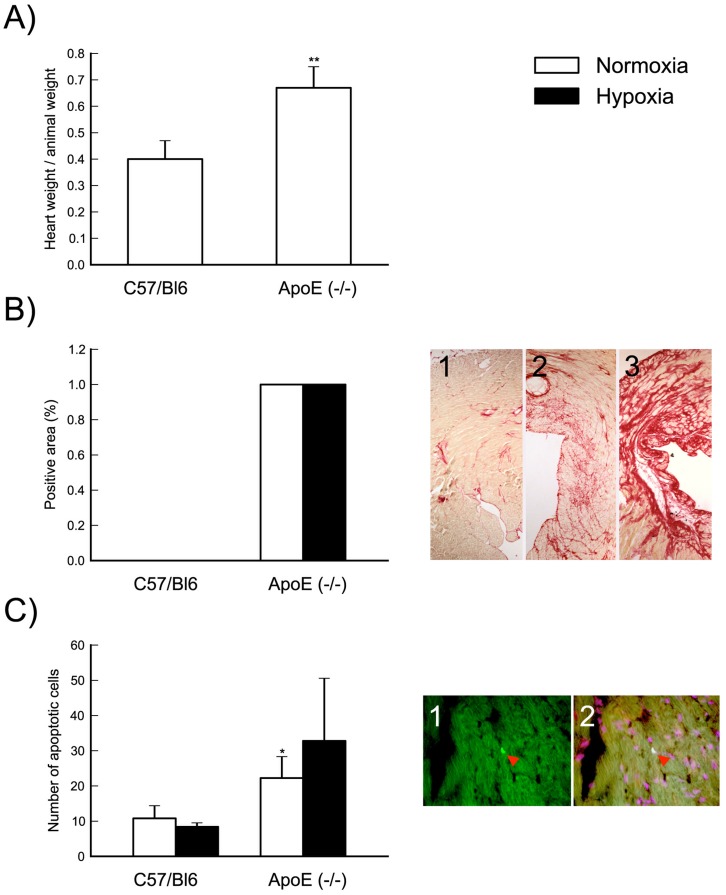
Apoe^−/−^ mouse as a model of atherosclerotic heart disease. Heart weight/animal weight ratios are significantly increased in Apoe^−/−^ mice compared to C57BL/6J (**, p<0,01 versus C57BL/6J) (Panel A). Hypercholesterolemic diet led to a strong increase of interstitial matrix collagen deposition in Apoe^−/−^ compared to C57BL/6J under both normoxic and hypoxic condition (Panel B left). We analyzed 3 coronal area sections from each animal and the results are expressed using a semiquantitative score: 1 absent collagen, 2 mild collagen deposition, 3 severe collagen deposition. Panel B right is a representative image of absent (1), mild (2) and severe (3) collagen deposition. The number of apoptotic cells is significantly increased in Apoe^−/−^ compared to C57BL/6J only under normoxic condition. We performed TUNEL staining on 3 coronal area sections from each animal and results are expressed as mean of number of apoptotic cells (*, p<0.05 versus C57BL/6J normoxia) (Panel C left). Panel C right is a representative image of positive TUNEL staining (1, arrow) and overlap with propidium iodide as a nuclei marker (2, arrow). Results presented are mean±SD of C57BL/6J normoxia n = 4; C57BL/6J hypoxia n = 5; Apoe^−/−^ normoxia n = 6; Apoe^−/−^ hypoxia n = 7.

It is well known that Apoe^−/−^ mice develop hypercholesterolemia and atherosclerotic lesions, including the coronaries [12] whereas wild type control mice (C57BL/6J) do not. Furthermore, recent reports indicate that Apoe^−/−^ show cardiac hypertrophy and interstitial remodeling [13]. When analyzing Apoe^−/−^ heart tissue we found similar functional and histopathological changes (Fig. 1). Thus, heart/body weight ratios were significantly increased in Apoe^−/−^ mice compared to C57BL/6J mice (Fig. 1A). Furthermore, a pronounced increase of interstitial matrix collagen deposition, detected with sirius red staining, is present in Apoe^−/−^ vs. C57BL/6J mice under both normoxic or hypoxic conditions (Fig. 1B). No evidence of necrotic areas were observed with hematoxylin/eosin staining and plasma levels of cardiac troponin I was not changed in C57BL/6J and Apoe^−/−^ mice after 1 year of a hypercholesterolemic diet under both normoxic and hypoxic conditions (data not shown). Therefore, no evidence of myocardial infarction could be demonstrated.

In addition, we analyzed if increased cell apoptosis correlated with atherosclerotic heart disease, applying a TUNEL staining procedure. TUNEL positive cells were considerably increased in Apoe^−/−^ compared to C57Bl6 mice (Fig. 1C), however, significant changes in apoptotic cell numbers could not be demonstrated in response to hypoxia in any of the mice models.

**Figure 2:**
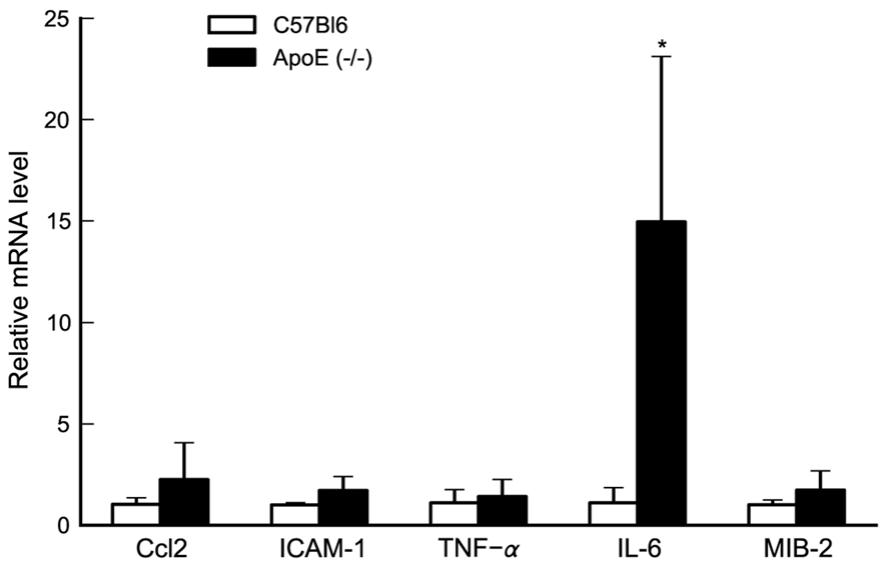
IL-6 expression is upregulated in cardiac tissue of Apoe^−/−^ mice. In the Apoe^−/−^ mouse model, baseline heart levels of IL-6 are increased as compared to control C57BL/6J mice whereas levels of TNF-a, Ccl2, ICAM-1, MIB-2 are unaltered. Each experiment was run in duplicate and changes in mRNA levels were expressed as ΔΔCt values and presented as relative to the mean of C57BL/6J mice. Values are mean±SD of C57BL/6J n = 4; Apoe^−/−^ n = 6. *, p<0.05.

Moreover, we found that the expression of IL-6 was robustly increased in Apoe^−/−^ mouse hearts as compared to wild type mice (p<0.05) whereas the expression levels of TNFα, CCL-2, ICAM-1, and MIB-2 were not significantly altered (Fig. 2). No significant additional changes in any of these cytokine levels were observed under hypoxic conditions in the myocardium, making it unlikely that these mediators were responsible for the effects of hypoxia. Thus, the fat-fed, aged Apoe^−/−^ mouse model develops severe atherosclerosis with myocardial hypertrophy, increased interstitial collagen deposition, increased apoptosis and a chronic increase in IL-6 levels without any evidence of myocardial infarction. We continued to specifically investigate the expression and role of the cys-LT system.

### LTC_4_S and CysLT1 Gene Expression is Up-regulated in Cardiac Tissue of Apoe^−/−^ Mice

**Figure 3:**
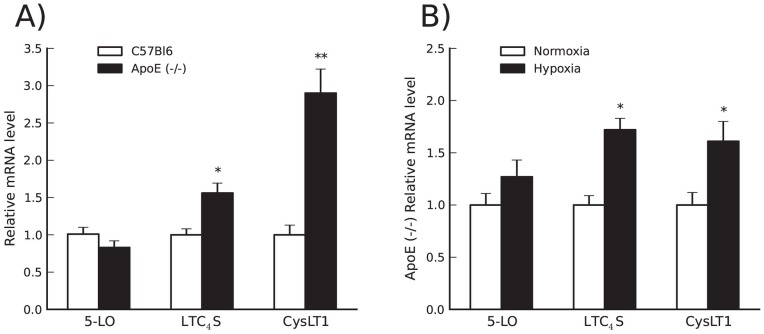
LTC_4_S and CysLT1 are upregulated in cardiac tissue of Apoe^−/−^ mice. In the Apoe^−/−^ heart, levels of LTC_4_S and CysLT1 are significantly upregulated as compared to control C57BL/6J mice (Panel A). Acute hypoxic stress in Apoe^−/−^ mice increases the cardiac expression of LTC_4_S (p<0.05) and CysLT1 (p = 0.06 for two sided t-test, p<0.05 for one sided t-test) compared to normoxic conditions (Panel B). Each experiment was run in duplicate and changes in mRNA levels were expressed as ΔΔCt values and presented as relative to the mean of C57BL/6J mice (Panel A) or normoxia (Panel B). Values are mean±SD of C57BL/6J normoxia n = 4; Apoe^−/−^ normoxia n = 6; Apoe^−/−^ hypoxia n = 7. *, p<0.05; **, p<0.01.

**Figure 4:**
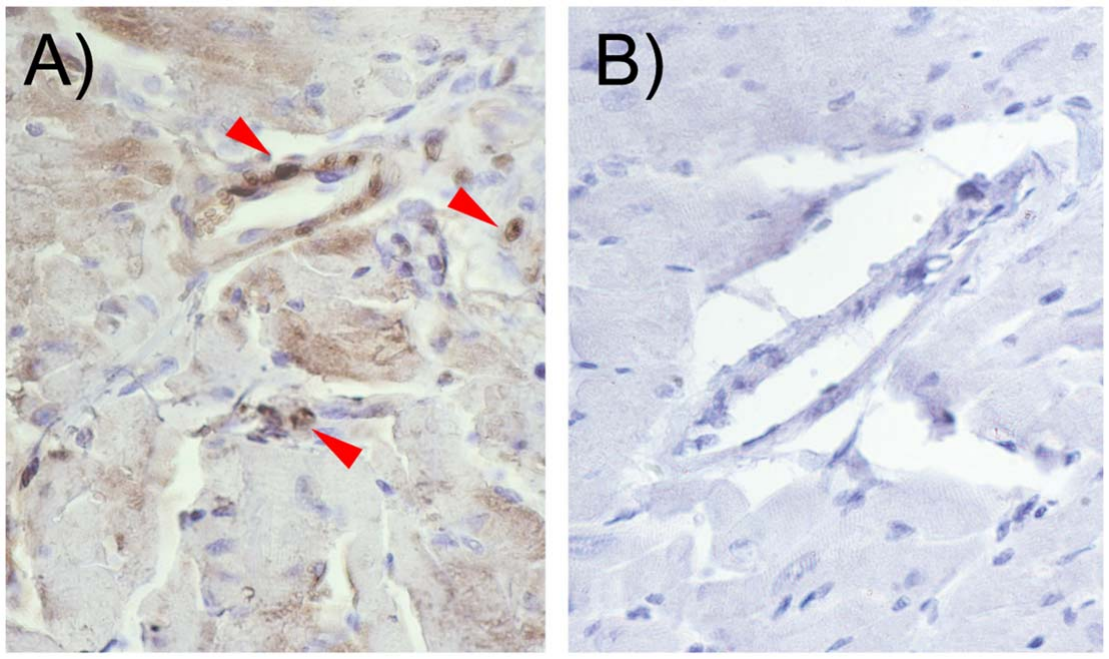
CysLT1 receptor is expressed in cardiac mouse tissue. Immunohistochemistry analysis confirms CysLT1 receptor expression in mouse heart tissue notably in endothelial cells. The panels show representative immunostaining for CysLT1 receptor expression in (A) Apoe−/− without hypoxia; (B) Apoe−/− following hypoxic stress and (C) negative controls. The arrows point to positive staining cells. The results are representative of staining performed in n = 6 per group.

We assessed the profile of LT cascade proteins at the mRNA level. In the Apoe^−/−^ heart, mRNA levels of LTC_4_S and CysLT1 were significantly upregulated as compared to control C57BL/6J mice whereas 5-LO levels remained unaltered (Fig. 3A). Immunohistochemical analyses confirmed the presence of CysLT1 receptor protein expression in mouse heart tissue in Apoe−/− mice without hypoxia and in Apoe−/− mice following hypoxic stress, mostly located in endothelial cells (Fig. 4).

### Hypoxic Stress Further Induces Expression of the cys-LT Pathway

Additional experiments were performed to determine the effects of acute hypoxic stress on LT pathway gene expression in Apoe^−/−^ mice. At 48 h after a bout of hypoxia a further increase in the expression of LTC_4_S (p<0.05) and CysLT1 (p<0.05) (Fig. 3B) was observed. In addition, we found a trend toward a further increase in the expression of 5-LO, the upstream enzyme in cys-LT biosynthesis (Fig. 3B).

### LTC_4_S Enzyme Activity is Increased in Cardiac Tissue of Apoe^−/−^ Mice

**Figure 5:**
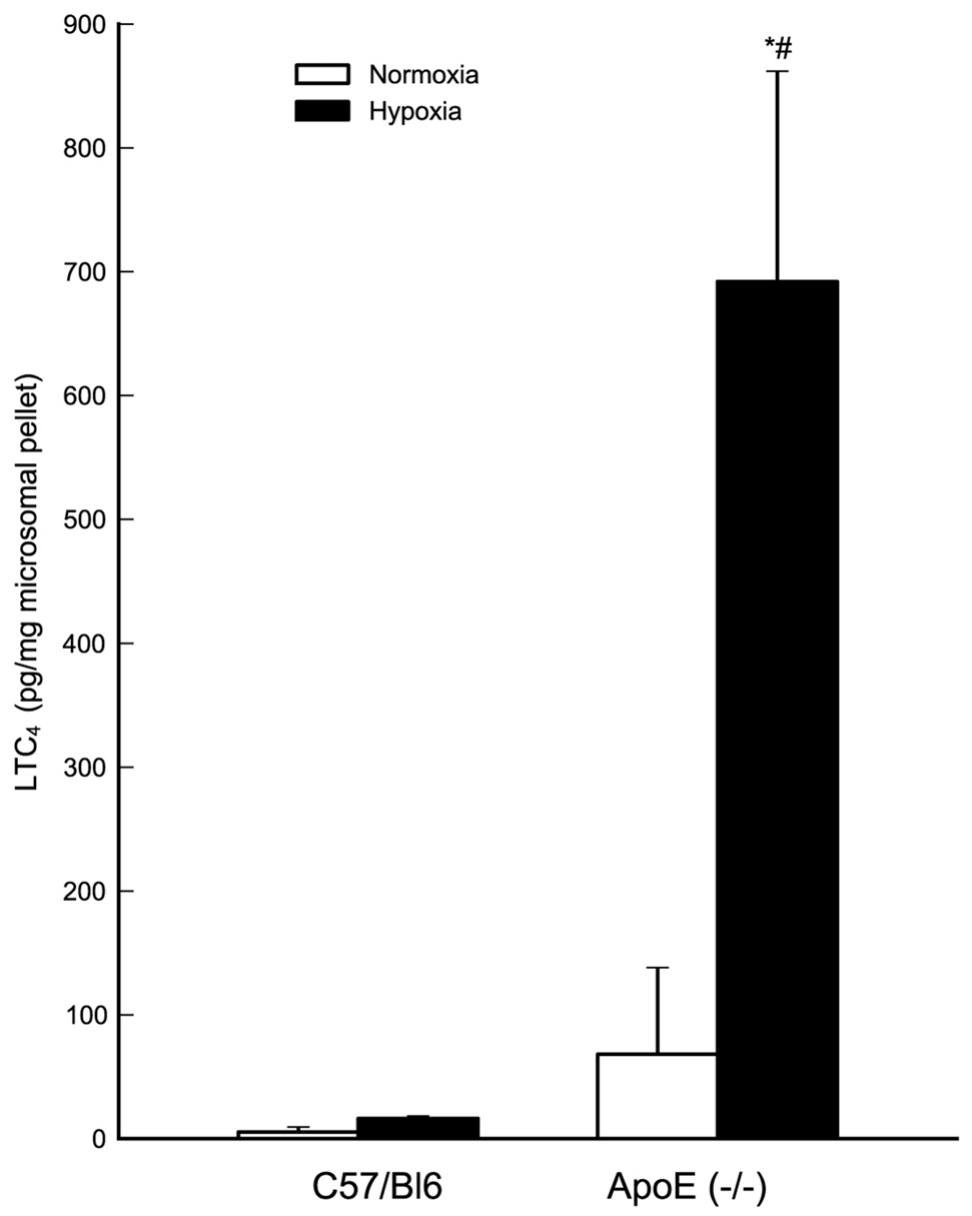
LTC_4_S enzyme activity is increased in cardiac tissue of Apoe^−/−^ mice. LTC_4_S enzymatic activity is enhanced in Apoe^−/−^ mice compared to C57BL/6J mice under both normoxic (not statistically significant) and hypoxic conditions (#, p<0.05 versus C57BL/6J hypoxia). LTC_4_S enzymatic activity was enhanced by 10-fold in Apoe^−/−^ mice under acute hypoxia compared to normoxia condition (*, p<0.05 versus Apoe^−/−^ normoxia). Results expressed as LTC_4_ (pg/mg microsomal pellet) are mean ± SD of C57BL/6J normoxia n = 3; C57BL/6J hypoxia n = 5; Apoe^−/−^ normoxia n = 4; Apoe^−/−^ hypoxia n = 3.

To detect LTC_4_S enzyme activity, microsomal preparations were isolated from Apoe^−/−^ heart tissue and incubated with intact LTA_4_, the key unstable intermediate of the LT cascade. Coupled HPLC/EIA analysis detected significant formation of LTC_4_, which increased in heart tissue from Apoe^−/−^ mice, particularly following hypoxic stress (Fig. 5).

### Blocking cys-LT Signaling Reduces Myocardial Hypoxic Load

**Figure 6:**
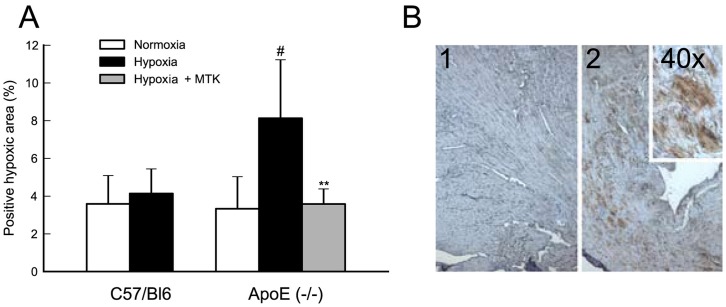
Blocking cys-LT signaling reduces myocardial hypoxic load. Hypoxic myocardial load is significantly upregulated under acute hypoxic stress in Apoe^−/−^ (#, p<0.05 versus Apoe^−/−^ normoxia); pretreatment with Montelukast (Hypoxia+MTK) reduces hypoxic myocardial load to levels equal to those observed in hearts of Apoe^−/−^ mice under normoxia (**, p<0.01 versus Apoe^−/−^ hypoxia) (Panel A). Panel B is a representative immunostaining for negative control (1) and positive staining (2) with 2 different magnifications. Hypoxyprobe signal was detected and quantified as described in the methods section. Results of Panel A are expressed as mean±SD of C57BL/6J normoxia n = 4; C57BL/6J hypoxia n = 5; Apoe^−/−^ normoxia n = 6; Apoe^−/−^ hypoxia n = 7; Apoe^−/−^ hypoxia with montelukast pre-treatment n = 7.

To evaluate the hypoxic load, mouse heart samples were stained with hypoxyprobe. Hypoxic myocardial load, expressed as % positive staining area, was significantly enhanced upon acute hypoxic stress in Apoe^−/−^ mice. In contrast, no changes were observed in the hypoxic myocardial area when C57Bl6 mice were subjected to acute hypoxia. When Apoe^−/−^ mice were pretreated with montelukast and subjected to an acute hypoxic stress, the hypoxic myocardial load decreased to basal normoxic levels (Fig. 6 A and B). It therefore seems likely that cys-LT signaling plays a more prominent role specifically in diseased tissue and not in healthy myocardium.

### Cys-LT Pathway Gene Expression in the Human Heart

**Figure 7:**
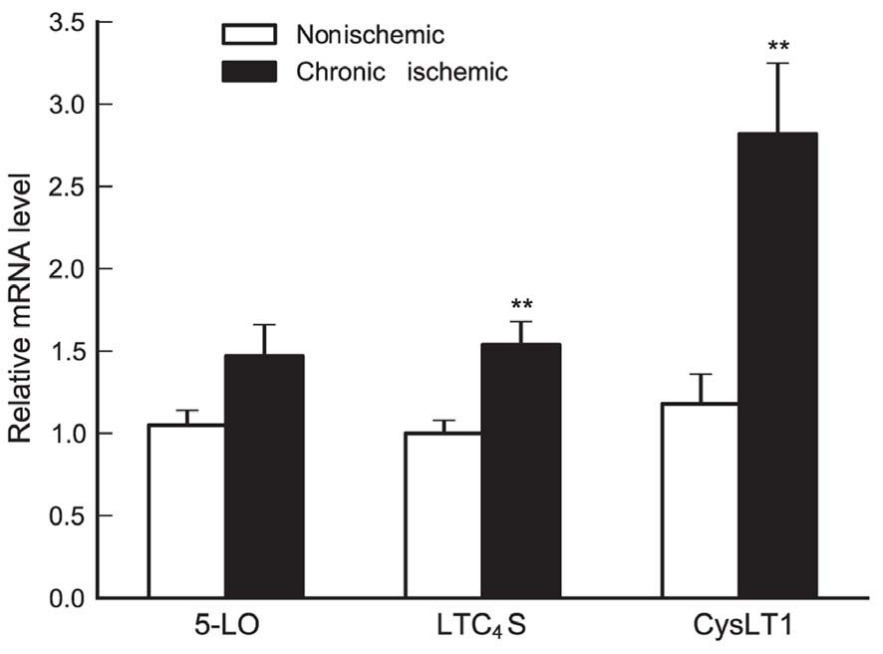
Cysteinyl-leukotriene signaling in human heart biopsies. In chronic ischemic myocardium, levels of LTC_4_S and CysLT1 are significantly upregulated as compared to non-ischemic myocardium. In each patient a heart biopsy from chronic ischemic area was compared with a control biopsy from the same patient in a non-ischemic area. Each experiment was run in duplicate and changes in mRNA levels were expressed as ΔΔCt values and presented as relative to the mean of non-ischemic myocardium. Values are mean ± SD of n = 14 patients. **, p<0.01.

We performed mRNA expression analyses of 5-LO, LTC_4_S and the CysLT1 receptor on a unique collection of human heart biopsies, obtained from chronic ischemic and non-ischemic parts of the same heart; i.e. each heart biopsy in chronic ischemic area was compared with a control biopsy from the same patient in a non-ischemic area. A significant increase in both LTC_4_S and CysLT1 mRNA levels was observed in chronic ischemic human myocardium (compared to non-ischemic myocardium) (Fig. 7), in agreement with the data obtained with Apoe^−/−^ mice (Fig. 3A).

## Discussion

Leukotrienes are potent pro-inflammatory lipid mediators and over the past years several studies have implicated LTs, in particular LTB_4_, in the inflammatory component underlying vascular inflammation, atherosclerosis, and atherothrombosis [8], [14], [15], [16], [17], [18]. The role of LTs in diseases related to the myocardium, however, remains unclear. Thus, in previous studies with preclinical models, hearts of otherwise healthy animals (rodents and dogs) have been subjected to ischemic damage and effects of genetic or pharmacological intervention of the leukotriene pathway have been studied with variable effects [19], [20]. In recent elegant studies, the second major receptor for cys-LTs, viz. CysLT2 has been implicated in vascular responses to cys-LT and ischemia-reperfusion injury [10], [21]. Here, a transgenic model was employed with artificial overexpression of CysLT2 in endothelial cells and, as in most previous investigations, the potential impact of pre-existing vascular disease or atherosclerosis was not addressed. Furthermore, is important to distinguish between models of ischemia/reperfusion with infarction and models of hypoxia without infarction. Accordingly, although studies have addressed the potential role of LTs in the setting of myocardial infarction and ischemia-reperfusion injury with influx of inflammatory cells (for review see [22]) understanding of the coupling between a hypoxic myocardial environment and induction of LT signaling is lacking. In particular, it is unclear whether and/or which of the molecular components of the LT system is expressed in the myocardium and if production of LTs occur. In addition, it is not known whether endogenous LT signaling elicits adverse effects in response to bouts of hypoxia. We therefore constructed an experimental system to test the hypothesis that cys-LT signaling may compromise the myocardial oxygen supply in chronic ischemic heart disease during bouts of hypoxia.

In the present study we chose the Apoe^−/−^ mouse as a model of atherosclerotic heart disease and assessed the gene expression profile of the LT cascade. The Apoe^−/−^ mouse develops hypercholesterolemia and extensive atherosclerotic lesions throughout the arterial tree as well as coronary atherosclerosis [12]. In addition, this mouse model exhibits cardiac hypertrophy and interstitial remodeling [13], findings that we were able to reproduce in this investigation (Fig. 1). We found that in the Apoe^−/−^ heart, levels of LTC_4_S and CysLT1 were specifically and significantly upregulated as compared to control C57BL/6J mice. Since LTC_4_S catalyzes the committed step in cys-LT formation and the CysLT1 receptor is known to mediate classical pro-inflammatory actions of cys-LT [23], this expression profile is consistent with increased cys-LT signaling in the heart in atherosclerotic disease.

Previous clinical investigations have demonstrated increased levels of urinary LTE_4_, a stable and bioactive metabolite of LTC_4_, in patients with sleep apnea [4] and acute coronary syndromes [7]. Moreover, treatment with a FLAP inhibitor suppressed biomarkers of infarction in a clinical trial of patients with a genetic variant of FLAP that confers increased risk of myocardial infarction [15]. However, these studies have measured systemic levels of LTs and none of them have presented direct evidence for a localized myocardial production of these mediators.

Furthermore, it is not previously known whether an acute hypoxic stimulus increases the expression of genes involved in LT production and signaling, which in turn would point to a possible link between hypoxia and inflammation in the heart. It was therefore of interest to assess the effects of a bout of hypoxic stress on LT-pathway gene expression. In our model, there was a pronounced increase in the expression of LTC_4_S and CysLT1 in response to hypoxic stress. In addition, we could detect a tendency towards increased expression of 5-LO, the upstream enzyme in LT biosynthesis. One could argue that increased expression of cys-LT system components are due to an increased influx of inflammatory cells in chronic hypoxic myocardium or following hypoxic stress. Although a contribution from recruited leukocytes cannot be entirely excluded it does not appear a likely explanation to our data, because we had no evidence of myocardial infarction by histology or troponin measurements and we counted inflammatory cells and found no differences in numbers of CD68+ cells.

CysLT1 protein was localized by immunohistochemistry to vascular (possibly endothelial) cells in heart tissue of Apoe^−/−^ mice. In the absence of a specific antiserum for LTC4S, we used a biochemical approach to demonstrate the presence of active enzyme in the mouse myocardium. Thus, incubation of heart membrane preparations with intact LTA_4_, followed by analysis with HPLC coupled to EIA, revealed significant formation of LTC_4_, which increased in heart tissue from Apoe^−/−^ mice, particularly following hypoxic stress. Because there is only one membrane enzyme with significant ability to conjugate LTA_4_ with GSH to form LTC_4_, *i.e.,* LTC_4_S, we conclude that this enzyme is up-regulated in hypoxic heart tissue from Apoe^−/−^ mice.

To test whether pharmacological interruption of the cys-LT signaling pathway could have an impact on the hypoxic load of the myocardium, Apoe^−/−^ mice were subjected to hypoxia in the presence or absence of montelukast, a selective antagonist of CysLT1. Administration of this drug reduced the hypoxic load of the myocardium to levels equal to those observed in hearts of Apoe^−/−^ mice under normoxic conditions. Since expression of LTC4S was also increased in the heart of Apoe^−/−^ mice, an alternative pharmacological approach to block cys-LT signaling could be inhibition of LTC_4_S [22]. This strategy may in fact be even more effective since recent data have shown that there are multiple, cross-talking, and cross-regulatory receptors for cys-LT [24], [25]. However, to date no selective LTC_4_S inhibitors have been developed. The choice of the classical CysLT1 antagonist montelukast in our study was guided by the selective upregulation of CysLT1 and the fact that montelukast is a widely prescribed asthma drug. Since both the endogenous ligands and the synthetic antagonists are known to display off-target actions, it had been desirable to study the effects of, e.g. modulatory GPR17 signaling, a selective LTC4S inhibitor, or additional CysLT1 antagonists. Due to the lack of sufficiently selective pharmacological tools and extended time needed for this mouse model, we focused on montelukast.

In studies of the pathological role of LTs, animal disease models have been of variable value. For instance, few, if any, mouse models reproduce human LT-driven asthma and for atherosclerosis, the Apoe^−/−^ model has provided ambiguous results [16], [26]. Therefore, it was of particular interest to compare our data from the Apoe^−/−^ model with human tissues. Needless to say, samples of human tissues that perfectly match the mouse model, or vice versa, are not available. However, we used a unique collection of human heart biopsies from both ischemic and non-ischemic parts of the same heart and could demonstrate similar changes in the expression levels of LTC_4_S and CysLT1 as those observed in the Apoe^−/−^ model (Fig. 7). These data suggest that the chronic ischemic human heart is also susceptible to increased cys-LT signaling and that blocking of cys-LT signaling may exert similar effects in human heart tissue under hypoxic stress. In this context it is interesting to note that a recent report demonstrates that males using montelukast have a significantly reduced risk of MI, supporting the clinical relevance of our work [27].

In conclusion, we have demonstrated that LTC_4_S and CysLT1 receptor gene expression is up-regulated in the heart and can be further enhanced by bouts of hypoxic stress in a mouse model of atherosclerotic heart disease. Blocking cys-LT signaling by montelukast abolishes the aggravated hypoxic load in response to hypoxia indicating that cys-LTs compromise oxygen supply, presumably via actions on the myocardial microcirculation. We also demonstrate a highly similar gene-expression pattern in the chronic ischemic human heart suggesting that similar mechanisms are operating in humans. These findings link myocardial hypoxia to inflammatory cys-LT signaling, vasoconstriction and acute hypoxic events, and prompts for testing of anti-leukotriene drugs in humans at risk for decreased blood oxygen content (e.g. sleep apnea) and with obstructive coronary artery disease.

## Materials and Methods

### Experimental Protocol

Animal procedures were approved by the Stockholm Ethical Committee on Animal Experiments (D.Nr. N28/07/N360/07). Male Apo-lipoprotein E-deficient [Apoe^−/−^] and C57BL/6J mice were fed a Western-type diet for 1 year. Apoe^−/−^ mice were treated with either vehicle i.p. dimethyl sulfoxide (DMSO) (n = 7, group A), montelukast 10 mg/kg i.p. (MTK) dissolved in DMSO (n = 7, group B) or left untreated (n = 6, group C). C57BL/6J mice were treated with vehicle i.p. (DMSO) (n = 5, group D) or left untreated (n = 4, group E). One hour after treatment, groups A, B, and D were exposed to hypoxic stress (10% O_2_) for 30 min. DMSO and montelukast treatment were repeated 24 h after hypoxic stress and animals were euthanized at 48 h. 30 min before euthanasia mice received pimonidazole hydrochloride (60 mg/kg i.p.; Hypoxyprobe, Chemicon International, Inc., Temecula, CA). Following perfusion with PBS, the hearts were collected and divided into thin slices and either embedded in OCT and flash-frozen or preserved in formaldehyde and paraffin-embedded. Cardiac troponin-I plasma levels were determined by ELISA (Life Diagnostics Inc., West Cheater, PA).

### Histochemical and Immunohistochemical Analyses

Myocardial sections were stained with hematoxylin eosin. Heart slices were also incubated with either Hypoxyprobe 1 Mab1 bound to FITC (diluted 1∶100, Chemicon International) for 1 h at room temperature or with CysLT1 receptor rabbit polyclonal antibody (diluted 1∶50, Cayman) overnight at 4°C. Hypoxyprobe signal was developed using anti-FITC/HRP complex (diluted 1∶200, Chemicon International), ABC-PO complex (Vectastain ABC Kit, PK-6100 standard, Immunokemi), and DAB substrate (Peroxidase substrate kit, SK-4100, Vector Laboratories) and counterstained with Mayer’s hematoxylin. Hypoxic area was quantified using Leica QWin Image analysis software (Leica, Wetzlar, Germany). CysLT1 receptor signal was developed with anti-rabbit biotinylated antibody (diluted 1∶500, PerkinElmer), streptavidin-HRP conjugated (diluted 1∶100, PerkinElmer), DAB substrate (Peroxidase substrate kit, SK-4100, Vector Laboratories) and counterstained with Mayer’s hematoxylin. Picrosirius red staining was performed on paraffin-embedded heart tissue sections for 1 hour in saturated picric acid containing 0.1% picrosirius red (Direct Red 80; Fluka, Buchs, Switzerland).

### Terminal Deoxynucleotidyl Transferase (TdT)-mediated dUTP Nick-end-labeling (TUNEL) Staining

The ApoAlert DNA Fragmentation Assay Kit (Clontech) was used to detect apoptosis-induced nuclear DNA fragmentation using a fluorescence assay according to the instructions by the manufacturer, and expressed as number of apoptotic cells per total area. Mouse thymus was used as TUNEL staining positive control.

### LTC_4_S Enzyme Assay

Heart microsomal fractions in aliquots of 85 µL, supplied with GSH (5 mM) and BSA (10 µg/µL), were incubated at RT for 10 min with LTA_4_ lithium salt (1 µM). Reactions were stopped with 2 vol. methanol containing 125 pmol of PGB_2_ as the internal standard, acidified to pH 5.6 [citrate (0.1 M)-Phosphate (0.2 M) buffer], and precipitated proteins removed by centrifugation (1000 x g, 4°C, 5 min). LTA_4_ transformation products were extracted on a Sep-Pak C18 cartridge (Oasis, Waters), samples eluted in 1 mL of methanol, dried under a N_2_ stream, reconstituted in methanol/water (1∶1) and analyzed by RP-HPLC coupled to enzyme-immunoassay (EIA kit, Cayman Chemical). Control LTA_4_ incubations with buffer alone or with recombinant LTC_4_S were run in parallel.

### Human Myocardial Biopsies

All subjects gave informed consent and the investigation was approved by the ethical committee of Copenhagen (KF 01–225/01) and the Karolinska Hospital (03–360). A total of 14 patients (Table 1) with coronary artery disease undergoing elective coronary artery by-pass grafting (CABG) were included in the investigation. Transmural “Tru-cut” biopsies (diameter 1–2 mm) were performed in each patient at 2 different sites of the left ventricle. The first biopsy site was chosen in an area with chronic ischemia due to a sub-occluded or occluded coronary artery, with no macroscopic signs of earlier infarction or fibrotic scar tissue. The second site was a myocardial region without ischemia.

**Table 1:** Clinical characteristics of patients.

Patients	*n* = 14
Age (years, mean ± SE)	68±2
Sex (M/F)	12/2
Diabetes, *n*	3
LVEF (%, mean ± SE)	55±3
Hyperlipidemia, *n*	11
Previous STEMI, *n*	6
Prior PCI, *n*	2
Prior CABG, *n*	0
CCS class (mean ± SE)	2.4±0.1
NTG, *n*	8
β-blocker, *n*	10
Ca2+ antagonist, *n*	5
Statin, *n*	12
Duration of acute myocardial ischemia (minutes, mean [min–max])	54 (28–100)
Time from reperfusion to biopsy (minutes, mean [min–max])	36 (20–75)
Coronary artery stenosis	
70–90%	14
90–95%	22
>95%	64
2-vessel disease (stenosis >70%), *n*	5
3-vessel disease (stenosis >70%), *n*	9

Values are expressed as number (*n*) of patients, mean ± standard error of the mean (SE) or mean with minimum (min) and maximum (max) values. LVEF, left ventricular ejection fraction determined by echo-cardiography; STEMI, ST-elevation myocardial infarction; PCI, percutaneous coronary intervention; CABG, coronary artery by-pass grafting; NTG, long-acting nitrate preparation.

### RNA Isolation, cDNA Synthesis, and Real-time Quantitative RT-PCR

RNA from left ventricular mouse heart tissue and human left ventricular heart biopsies was isolated with RNeasy mini kit (Qiagen) according to the manufacturer’s instructions. Quantity and quality was controlled using an Agilent Bioanalyzer 2100 (Agilent Technologies, Palo Alto, CA, USA). Complementary DNA (cDNA) was reverse transcribed from 0.5–1 µg of total RNA using random hexamer primers. Quantitative real-time PCR was performed on an ABI Prism 7700 sequence detector (Applied Biosystems, Foster City, CA, USA) with primer and probes purchased from assay-on-demand (Applied Biosystems). Changes in mRNA levels were expressed as ΔΔCt using Cyclophilin A as endogenous control [28].

### Statistical Analysis

Two sided parametric t-test, two-way ANOVA with a post hoc Holm-Sidak test was used to detect statistically significant differences (p<0.05) unless otherwise indicated. Data are expressed as mean ± standard deviation (SD) unless otherwise indicated.
